# A Case Report of Esophageal Bronchogenic Cyst and Review of the Literature With an Emphasis on Endoscopic Ultrasonography Appearance

**DOI:** 10.1097/MD.0000000000003111

**Published:** 2016-03-18

**Authors:** Chaoqun Han, Rong Lin, Jun Yu, Qin Zhang, Yang Zhang, Jun Liu, Zhen Ding, Xiaohua Hou

**Affiliations:** From the Division of Gastroenterology (CH, RL, JL, ZD, XH); Department of Pathology (JY, QZ); and Department of Thoracic Surgery (YZ), Union Hospital, Tongji Medical College, Huazhong University of Science and Technology, Wuhan, China.

## Abstract

Esophageal bronchogenic cysts are extremely rare. Here we report a more rare type of both presence of intra- and paraesophageal bronchogenic cyst that was safely removed via surgical resection. A 31-year-old male patient with space-occupying lesions in the mediastinum suddenly presented with persistent chest pain for 2 days and then transferred to dysphagia >1 week. Preoperative diagnosis is difficult. Endoscopic ultrasonography (EUS) showed a hypoechoic cystic-solid mass arising from the muscularis propria and local hyperechoic area in the deeper portion of cyst, concomitant with a heterogeneous center and tube-like structure lesion in mediastinum. Turbid coffee color paste contents were aspirated inside the tumor under endoscopic ultrasonography guided-fine needle aspiration (EUS-FNA). A subsequent surgery was performed and histologic finding was diagnostic of esophageal bronchogenic cyst. Immunohistochemical staining confirmed the cyst was positive for carbohydrate antigen 199 (CA199) and carbohydrate antigen 125 (CA125). At a follow-up visit 3 months later, the patient had a regular diet and no complaint. This study is to summarize the clinical manifestations and EUS features of esophageal bronchogenic cyst by retrospectively reviewing the literature and simultaneously to provide guide for the correct examination scheme.

The appearance of esophageal bronchogenic cyst can be great variation; EUS seems to be a valuable option for diagnosis and surveillance.

## INTRODUCTION

Bronchogenic cysts are rare congenital malformation originated from the development of ventral foregut, and mostly located in the middle and superior mediastinum.^1^ They more frequently situated in close relation to tracheobronchial tree or lung parenchyma with or without a well-defined borderline.^2,3^ Although these lesions are often benign, bronchogenic cysts can also cause symptoms such as chest pain, cough, dysphagia, and dyspnea via compression of the surrounding structures.^4^ As the majority of cysts are within the mediastinum, previous para-esophageal bronchogenic cysts have been frequently reported. Sporadic cases concerning intramural esophageal bronchogenic cysts are reported.^5^ Moreover, despite improved imaging modalities, a definitive preoperative diagnosis is difficult. To our knowledge, this is the first report to share our experience with a very rarely clinical entity of both periesophageal and intramural esophageal bronchogenic cysts with positive expressions of CA199 and CA125 by immunohistochemical study and also review of the literature with an emphasis on endoscopic ultrasonography image characteristics to guide correct examination scheme. (The answer to Reviewer #1[1] and [2].) Patients have signed informed consent and data had been anonymized and deidentified.

### Case Presentation

**FIGURE 1:**
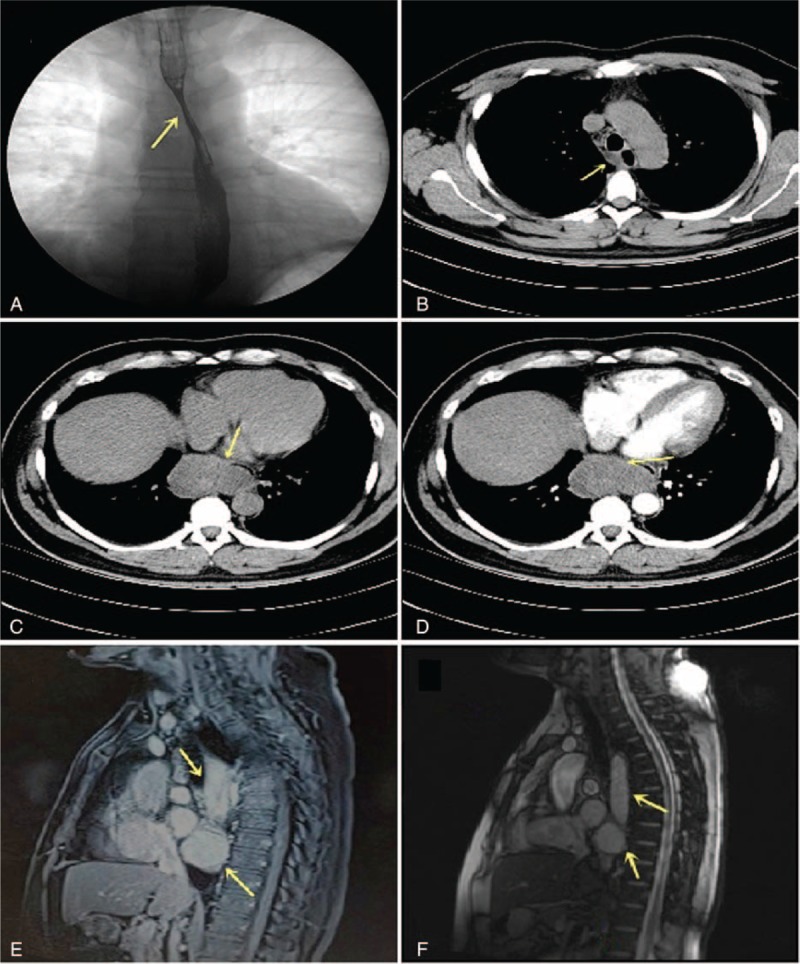
Preoperative imaging diagnoses. (A) A barium swallow identified an extrinsic compression shadow ∼13 cm in length under the aortic arch (arrowheads), with a regular mucosal pattern. (B) The esophageal computer tomography revealed a sharply defined mass attached to the right side of esophageal wall. (C) The maximum cross-section area was 7.2 × 4.3 cm and presented dumbbell-shaped (arrowheads). (D) Parenchyma was heterogeneous with slight enhancement on contrast material-enhanced CT scan. (E,F) Magnetic resonance imaging (MRI) images show a large circumscribed (a maximum cross-section area was 4.5 × 7.3 cm) tubular cystic lesion and a round mass behind the trachea, concomitant with compressed esophagus. The masses had a relatively high-intensity on both T1- and T2-weighted images. CT = computerized tomography, MRI = magnetic resonance imaging.

**FIGURE 2:**
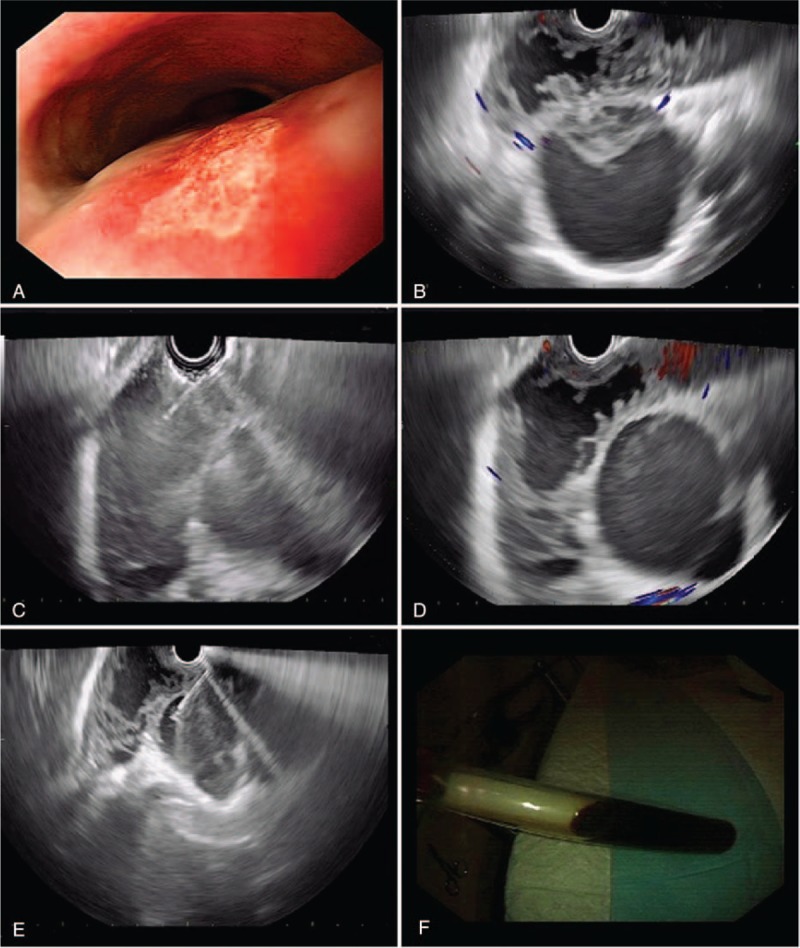
The characteristics of EUS appearance. (A) Upper endoscopy revealed a large ovoid bulging mass, 2.5 cm in diameter, with an erosion surface in the right anterior wall of the esophagus (arrowhead). (B–E) EUS evidenced that proper muscularis propria of the esophagus was damaged and tumor parenchyma was hypoechoic cystic-solid, concomitant with a heterogeneous center and tube-like structure lesion in mediastinum. (F) Concentrated coffee color fluid contents were aspirated by EUS-FNA. EUS = endoscopic ultrasonography, EUS-FNA = endoscopic ultrasonography guided-fine needle aspiration.

A 31-year-old man who presented to our hospital with suddenly symptoms of persistent chest pain for 2 days and then transferred to dysphagia >1 week. He has no fever, cough, vomit, and weight loss. And there was no obvious positive characteristics on physical exams. With regard to laboratory values, only serum tumor marker CA125 (>1000 U/mL; normal reference range: <35 U/mL) and CA199 (156.3 U/mL; normal reference range: <37 U/mL) levels were remarkable increased. Blood routine test, erythrocyte sedimentation rate (ESR), C-reactive protein, hepatic and renal function were all within normal limits. An esophagography obtained before presentation identified a huge filling defect compressed to esophagus under the aortic arch (Figure 1A). The thoracic computer tomography (CT) scanned a 14.5 × 2.3 cm ovoid and well-defined low-density mass located in the right posterior mediastinum, with a maximum cross-section area of 7.2 × 4.3 cm and presented dumbbell-shaped (Figure 1B–D). Magnetic resonance imaging (MRI) also showed a cystic lesion by relatively high-signal intensity behind the trachea and with a direct displacement of the esophagus (Figure 1E and F). In addition, gastroendoscopy and endoscopic ultrasonography were also performed to clarify anatomic relations. At esophagoscopy, a large semispherical swelling covered by some mucosal erosion was noted at 25 cm from the incisors to the dentate line (Figure 2A). Endoscopic ultrasonography (EUS) with 7.5 to 12 MHz showed a diameter of 25 mm hypoechoic cystic-solid mass arising from the muscularis propria and local hyperechoic area in the deeper portion of cyst, concomitant with a heterogeneous center and tube-like structure lesion in mediastinum (Figure 2B–E). Turbid coffee color paste contents were aspirated inside the tumor under EUS-FNA (Figure 2F). Malignant cells were not detected on cytopathologic evaluation. Surprisingly in our case, the CA125 value of the intracystic fluid was measured as>1000 U/mL with an increased level of CA199 (>1200 U/mL). With these findings, a diagnosis of muscularis propria and mediastinal cysts were highly suspected and surgical exploration was planned due to the intolerable symptoms.

**FIGURE 3:**
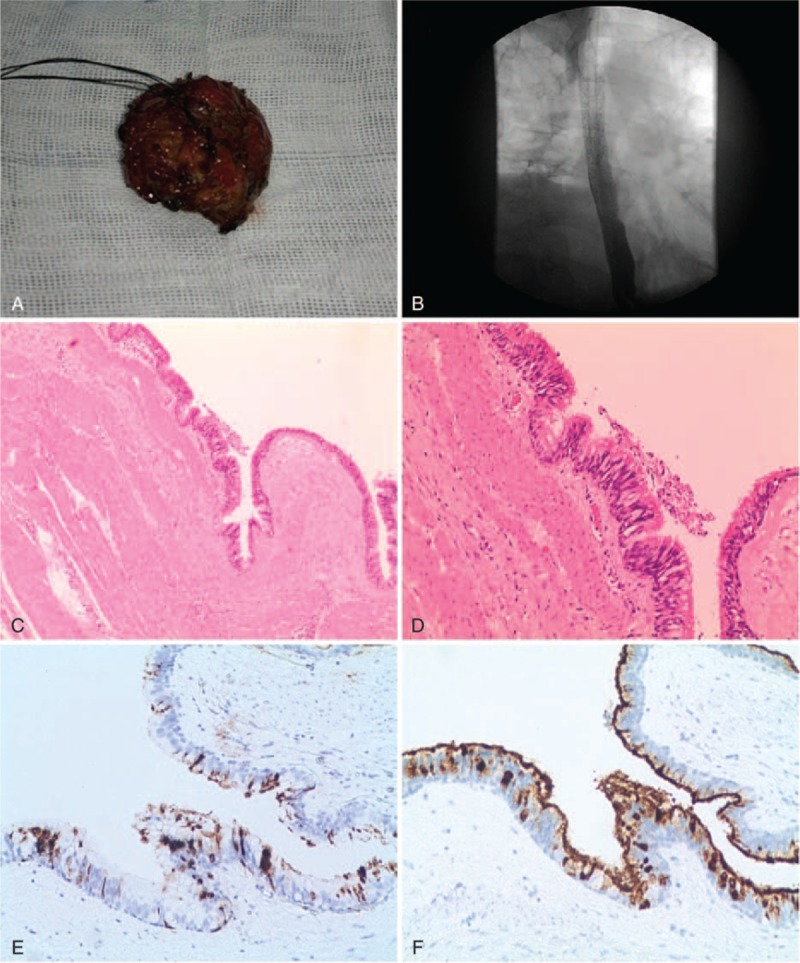
Complete removal of the paraesophageal cyst through a right thoracotomy and pathological results. (A) At macroscopic appearance, a 5 cm × 5 cm smooth-walled large cyst was filled with coffee color mucus. (B) Postsurgical esophagogram showing there was no communication with surrounding structures. (C) Microscopically, bronchogenic tissue including ciliated columnar epithelium lining and smooth muscle layers in the cyst wall but no cartilage or glands (hematoxylin and eosin [H&E]; magnification ×100). (D) Close-up of ciliated columnar epithelium lining of the cyst (H&E; magnification ×200). (E) Positive expression of CA199 in cyst sections by immunohistochemical staining, original magnification ×100. (F) Positive expression of CA125 in cyst sections by immunohistochemical staining, original magnification ×100. H&E = hematoxylin and eosin.

The patient underwent a right thoracotomy on the sixth intercostal space and 2 cysts were found. Macroscopically, one cyst was well demarcated, situated in lower pare-esophageal near cardia of stomach and extraction completion (Figure 3A). However, the length of other cyst was exceed 10 cm concomitant with the esophagus and located in the esophageal muscularis propria. Considering the lesion is too long and complete resection could affect esophageal dynamic function, it was decided to perform a thoracentesison-Tube drain for external drainage. Both cysts had an intact inner surface, without any communication with the esophageal lumen or adhesion to the adjacent structures. Ciliated columnar epithelium lining the cyst wall with underlying smooth muscles were found in the microscope and the diagnosis of esophageal bronchogenic cyst was finally confirmed (Figure 3C and D). We also found that positive expression of CA199 and CA125 in cyst sections by immunohistochemical staining (Figure 3E and F). The patient's intra-operative and postoperative evolution was uneventful with a rapid recovery. An esophageal iodine oil contrast at the seventh day after surgery was performed in order to guarantee esophageal lumen was no leakage (Figure 3B). At a follow-up visit 3 months later, the patient had a regular diet and no complaint.

## DISCUSSION

Bronchogenic cysts are broncho-pulmonary foregut abnormalities that usually occur along the tracheobronchial tree in the mediastinum. They may be located in paratracheal, carinal, or hilar.^6,7^ However, esophageal bronchogenic cyst is extremely rare and morbidity is still unknown due to most patients asymptomatic.^8^ When present, the symptoms are usually caused by compression of the surrounding structures or by complications related to the cyst^9^ (The answer to Reviewer #2[2]). Here we reported a more rare type of both presence of intra- and para-esophageal bronchogenic cyst that was safely removed via surgical resection. To our knowledge, a similar case had never been reported.

Different imaging techniques can be used for preoperative diagnosis and differential diagnoses included a duplication cyst, esophageal leiomyoma, pleural fibroma, lymphadenopathy, and bronchogenic cyst.^10^ Despite improved imaging modalities, a definitive diagnosis remains difficult preoperatively. CT or MRI sometimes can indicate the nature of the cyst. But once bronchogenic cyst is infected or high in protein or calcium content, its density may fall into the same solid mass scope that increases diagnostic uncertainty.^11,12^ Moreover, the intramural and extramural relationship of esophageal cysts is also difficult to identification. EUS is sensitive for distinguishing cystic from solid masses and facilitates the diagnosis of intramural esophageal lesions because of its capacity to clearly delineate the size, layer of origin, and the relationship of the cyst to the esophagus.^3^ The present study first to review of the literature with an emphasis on EUS appearance of esophageal bronchogenic cyst, as only 8 cases have been presented in the literature since 2000s^12–17^ (Table 1). The structure of the cyst can be round, oval or tubular, which mostly originates from muscularis propria. The appearance can range from being hypoechoic to containing dense hyperechoic debris which potentially confused with soft lesions. In our case, EUS clearly demonstrated a hypoechoic cystic-solid tumor that muscularis propria was damaged and local hyperechoic area in esophagus, as well as defined the extramural extent of lesions in mediastinum.

**TABLE 1:**
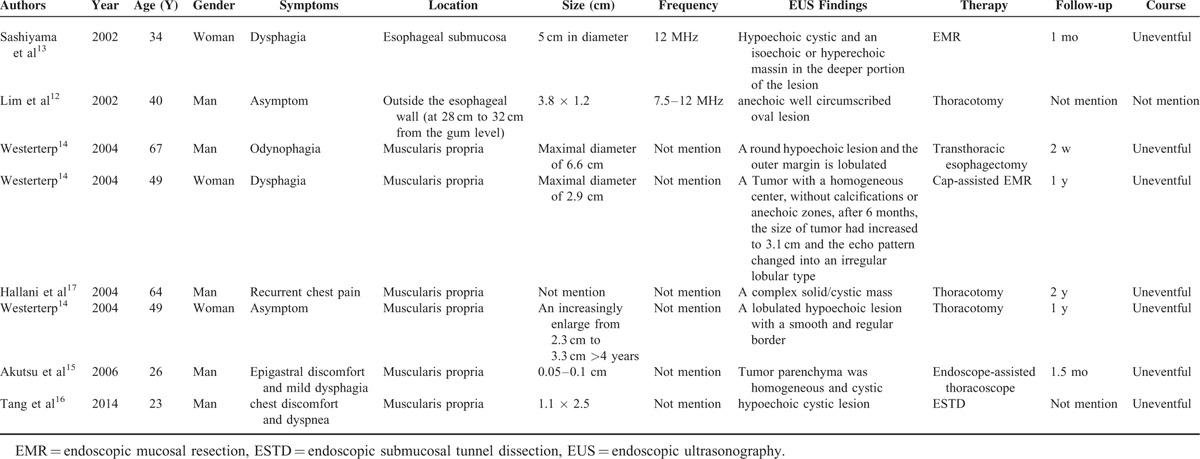
Endoscopic Ultrasonography Features of Published Cases With Periesophageal or Intramural Esophageal Bronchogenic Cyst

Because of great variation in the EUS appearance and severe consequences, the routine application of EUS-FNA in bronchogenic cysts is not advocated;^18^ however, in atypical and uncertain mediastinal cystic masses, it is necessary to perform FNA to obtain samples for a definitive cytological/histological diagnosis. Concentrated coffee color fluid was aspirated by EUS-FNA in our report. Both of serum and intracystic fluid levels of CA125 and CA199 were found to be remarkably elevated. CA125 is the main marker of choice for ovarian carcinoma and uncommonly reported in bronchogenic cyst,^19^ especially in esophageal bronchogenic cysts, reports of high levels of both CA199 and CA125 are extremely rare.^13,20^ We also first confirm CA199 and CA125 are positive expression in cyst sections by immunohistochemical staining. Measurement of CA199 and CA125 levels may be a diagnostic implication when we encounter a bronchogenic cyst of esophagus.

With regarding to the management of esophageal bronchogenic cysts, complete surgical removal by thoracotomy or video-assisted thoracoscopy is recommended, even when they are asymptomatic, because of subsequent complication of infection, rupture, intracystic hemorrhage, carcinomatous change.^21,22^ As in our case, the intramural cyst may be associated with greater difficulty to resection due to the lesion length. Endoscopic submucosal tunnel dissection (ESTD) is a newly effective and safe procedure to treat submucosal tumors originating from the muscularis propria and has been successful to extract an intraesophageal bronchogenic cyst .^16,23^ This treatment would be a less complicated and less risky choice, but long-term follow-up visits and complications are required to evaluated further, EUS seems to be a valuable option for diagnosis and surveillance.

## AUTHOR CONTRIBUTIONS

Chaoqun Han: collection and assembly of data, data analysis, manuscript writing, performed the literature search; Rong Lin: collection and assembly of data, manuscript writing and performed the literature search; Jun Yu: provision of pathological data and valuable suggestions; Qin Zhang: provision of pathological data and valuable suggestions; Yang Zhang: provision of gross samples post operation; Jun Liu: administrative support and valuable suggestions; Zhen Ding: conception and design, financial support, revised the manuscript as corresponding author; and Xiaohua Hou: financial support and valuable suggestions.
